# Approximation to the Consumption of Healthcare Resources and the Economic Cost of SARS-CoV-2 Patient Management: A Retrospective Study

**DOI:** 10.3389/fpubh.2022.843751

**Published:** 2022-03-31

**Authors:** Jesús Calderón-Moreno, Raúl Juárez-Vela, María Jesús Delgado-Rodríguez, Manuel Quintana-Díaz, Rosa Magallón-Botaya, Bárbara Olivan-Blázquez, Ana Cobos-Rincón, Iván Santolalla-Arnedo, Carmen Amaia Ramírez-Torres, Vicente Gea-Caballero, Eva María Andrés-Esteban

**Affiliations:** ^1^Business Economics Department, University of Rey Juan Carlos, Madrid, Spain; ^2^Research Group Blood Patient Management, IDI-Paz Research Institute, Madrid, Spain; ^3^Department of Nursing, GRUPAC, University of La Rioja, Logroño, Spain; ^4^Intensive Care Unit, Research Group Blood Patient Management, IDI-Paz Research Institute, University Hospital “La Paz, ” Madrid, Spain; ^5^Primary Care Prevention and Health Promotion Network, Research Unit in Primary Care, IIS Aragón, University of Zaragoza, Zaragoza, Spain; ^6^Department of Psychology and Sociology, Institute for Health Research Aragón (IISA), University of Zaragoza, Zaragoza, Spain; ^7^Faculty of Health Sciences, Valencia International University, Valencia, Spain

**Keywords:** health economics, COVID-19, SARS-CoV-2, coronavirus infections, cost control and cost management

## Abstract

Spain has become one of the countries most affected by coronavirus disease 2019 (COVID-19), with the highest testing rates, and one of the worst-performing countries in the fight against the severe acute respiratory syndrome coronavirus 2 (SARS-CoV-2) pandemic. There are no studies related to the consumption of health resources and the economic cost of the SARS-CoV-2 virus. We present a retrospective analysis of 9,811 (Primary Care and Hospital) patients which aimed to estimate public health expenditure by the consumption of health resources due to COVID-19. According to the results, the gender distribution of patients has a similar rate in both groups, with slightly higher rates in women. Similarly, age is the same in both groups, with a median of 62 years in the case of hospitalizations and 61 years in the case of primary care; using a weighted average of these rates and costs, we can estimate that the average cost of care per patient infected with the SARS-CoV-2 virus, regardless of the course is €2373.24. We conclude that a patient with COVID-19 without hospitalization costs €729.79, while the expenses of a hospitalized patient are between €4294.36 and €14440.68, if there is ICU admission.

## Introduction

The Spanish National Health System (SNS) is based on a Beveridge-type public model (1). It is a decentralized national health system, with competencies transferred to 17 Spanish autonomous communities (regions), under the control of the Ministry of Health. Spanish autonomous communities have been crucial in the economic support of the health system in the coronavirus disease 2019 (COVID-19) pandemic, as they manage health spending. The SNS provides three levels of care: primary, specialized, and partner-health. The SNS coverage gradually spread until 100% of citizens were covered in 1989. Today, care is financed by taxes and services are accessed by health cards.

Despite universal health care coverage for the Spanish population, Spain has become one of the countries most affected by the disease with the highest COVID-19 testing rates (2–4) and one of the worst-performing countries in the fight against the severe acute respiratory syndrome coronavirus 2 (SARS-CoV-2) pandemic. Specifically, the country is fifth among nations with the highest population mortality rate (4), placing itself at the top of the OECD (Organization for Economic Cooperation and Development) countries list based on the proposed performance indicators for COVID-19 (5).

This situation has had to be faced in difficult economic circumstances. Investment in the SNS has been decreasing since 2009 until it stabilized at investment figures of ~5.9-6.1% in recent years. According to the Ministry of Health of Spain (6), in 2019 the consolidated expenditure of gross domestic product (GDP) was 6.0%, much lower than other European countries such as Germany (9.7%), France (9.4%), Sweden (9.3%), or the United Kingdom (7.8%). Moreover, spending by region has been uneven, with autonomous communities investing up to €1,753 *per capita* (Basque Country), and others with only €1,312 (Andalusia) or €1,274 (Madrid) (7). The SNS's “low” health expenditure is one of the causes of the poor response to our country's COVID-19 crisis (8), considering the post-global economic crisis “austerity” of 2008 as an aggravating factor of the “low economic capacity” (9).

There are no studies related to the consumption of health resources and the economic cost of the SARS-CoV-2 virus. We can only find some global data through entities like FAIR Health. The average cost of hospital care for COVID-19 patients varies enormously by age—from $51,389 for patients between 21 and 40-years-old to $78,569 for patients between 41 and 60 years old, according to the updated cost analysis data from FAIR Health (7–9). These economic costs can pose a serious threat to countries with tight budgets in their National Health System, such as Spain, with very high incidences and prevalence of confirmed cases, and with very high cumulative incidences in the form of “waves”^1^ (10, 11); currently, almost 3.5 million people have been positive, with more than 77,500 deaths. In order to respond to the economic aspects related to healthcare systems and the COVID-19 pandemic, it is necessary to generate greater knowledge on the consumption of healthcare resources and the economic costs associated with it. This knowledge will allow managers and governments to allocate resources in a more realistic way. However, we have found no data in our country that show the consumption of health resources used to care for patients with COVID-19 and who left the Spanish health system in a real collapse during the period March-June 2020 (first COVID-19 wave) and much less the cost of managing the patient infected with the SARS-CoV-2 virus that has brought the government of the country.

We presented a retrospective analysis of 9,811 patients to estimate public health expenditure by the consumption of health resources due to COVID-19.

## Methods

### Study Design and Participants

We conducted a retrospective cohort study that considered demographic data, previous comorbidities, and care variables of two populations: primary care population and hospital population. Regarding primary care, we obtained 6,286 patients diagnosed with COVID-19 in Aragon (Spain) from the beginning of the current pandemic to 30 June, 2020. In the case of the hospital population, a total of 3,525 clinical episodes were extracted from the database of 3,112 patients from the “Hospital Clinico Universitario La Paz (HULP)” during the same period. For the inclusion criteria, we analyzed all patients, over 18 years old, with COVID-19 based on the detection of SARS-CoV-2 RNA in throat swab specimens.

### Main Variables

We extracted all data on the demographics, clinical treatments, and outcomes from electronic medical records. Data were collected on the age, sex, and origin of the patient; total costs according to the care setting (primary care, admission to hospital ward, admission to ICU); the number of X-rays and number of CAT scans, and costs derived from the number of X-rays and CAT scans; costs according to the most frequent complications (respiratory infection, pneumonia, acute respiratory distress syndrome, pneumothorax, pleural effusion, meningitis, convulsions, stroke, heart failure, endocarditis, arrhythmia, cardiac ischemia, cardiac arrest, coagulation problems, anemia, renal failure, pancreatitis, hepatic failure, psychiatric illness, and gastrointestinal bleeding); as well as variables associated with chronic health problems that have been associated with complications in COVID-19 (smoking, diabetes, hypertension, chronic cardiac diseases, asthma, or chronic bronchitis).

Procedures and statistical analysis quantitative variables were presented using robust statistics, such as mean and interquartile ranges (IQRs), and qualitative data were presented using their frequency distribution. For the comparison of quantitative data between groups, we employed the Kruskal–Wallis non-parametric *H*-test and the Shapiro–Wilk test for non-normally distributed variables. All analyses were calculated using STATA/SE v16.0 and *p*-values lower than 5% were considered statistically significant.

The costs of patient management in public hospitals are published in the Spanish Official State Gazette.

## Results

The description of both cohorts, primary care, and specialized care (hospital) are shown in Table 1. The gender distribution of patients has a similar rate in both groups, with slightly higher rates in women. Similarly, age is the same in both groups, with a median of 62 years in the case of hospitalizations and 61 years in the case of primary care. The origin of the patients was similar in both groups too.

**Table 1:** Demographic characteristics of both cohorts.

	**Hospital population**		**Primary care population**	
**Characteristics**	**Frequency**	**%**	**Frequency**	**%**
**Gender**				
Men	1,725	48.94%	2,738	43.56%
Women	1,800	51.06%	3,548	56.44%
Age (median, IQRs)	62 (47-78)		61 (45-82)	
**Housing**				
Uncrowded house conditions	3,163	89.73%	6,039	96.07%
Nursing homes	314	8.91%	181	2.88%
Shelter residences	13	0.37%	56	0.89%
Prison	1	0.03%	0	0.00%
Other	34	0.96%	10	0.16%

**Figure 1:**
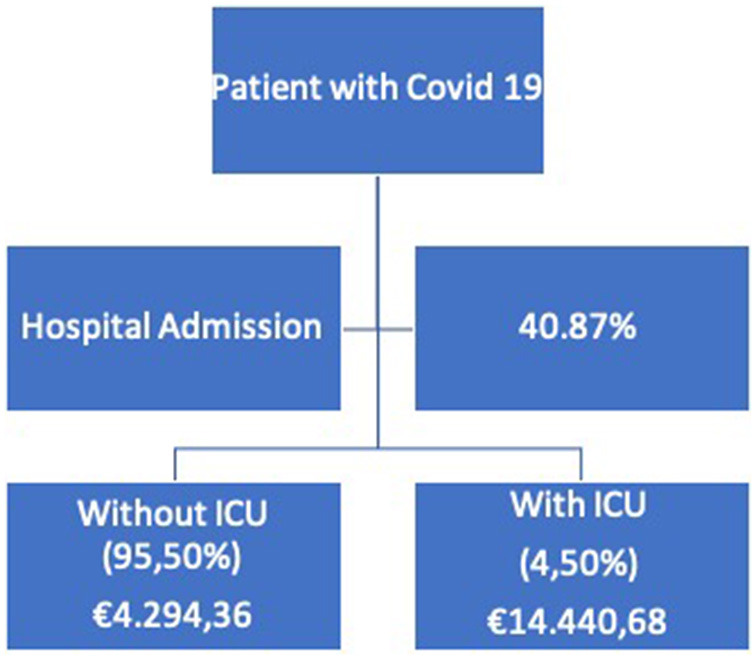
Decision tree.

With regard to the cost per visit, Figure 1 shows a decision tree of what happened in the first wave of the pandemic. In this regard, we see that only 59.13% of patients were seen exclusively in primary care, including visits to the health center, home visits, or patient telephone care, with an average associated cost of €729.79 per patient. The rest (40.87%) had to go to the hospital emergency department. The cost of the emergency department is equivalent to the cost of the ward per day, i.e., those patients who only went to the emergency department but were not hospitalized were counted as a day of specialized care. In this sense, there are two clearly differentiated scenarios: the cost of hospitalization on the ward, which averaged €4294.36 per patient, and the cost of hospitalization in the ICU, which amounted to €14440.68 per patient. Finally, using a weighted average of these rates and costs, we can estimate that the average cost of care per patient infected with SARS-CoV-2 virus, regardless of their course, was €2373.24 per person.

Concerning imaging tests, the following table (Table 2) shows the distribution of the number of X-rays and CAT scans performed on patients, as well as the cost involved. In our sample, a total of €95766.66 in X-rays and €41964.80 in CAT scans was spent, corresponding to €27.17 and €11.90 per patient, respectively.

**Table 2:** Cost of diagnostic imaging.

	**Patients**	**Total cost**
**Number of X-rays**		
0	448 (12.81%)	0
1	1989 (56.90%)	43,320.42 €
2	841 (24.06%)	36,633.96 €
3	160 (4.57%)	10,454.40 €
4	46 (1.31%)	4,007.52 €
5	8 (0.22%)	871.20 €
6	1 (0.02%)	130.68 €
7	0 (0%)	0.00 €
8	2 (0.05%)	348.48 €
**Number of CAT**		
0	3,169 (90.67%)	0.00 €
1	320 (9.15%)	40,448.00 €
2	6 (0.17%)	1,516.80 €

The cost of managing complications due to SARS-CoV-2 infection in our sample was €1707.66 per patient. Kidney failure, respiratory infection, and acute respiratory distress syndrome were the most frequent and most costly complications. Table 3 shows the cost per patient and the incidence of each of the complications considered in the study.

**Table 3:** Cost of complications.

**Complications**	**Cost per patient**	**Incidence**
Kidney failure	4,693.87 €	8.46%
Respiratory infection	3,495.68 €	7.54%
Acute respiratory distress syndrome	3,364.29 €	6.45%
Pneumonia	2,625.36 €	4.16%
Coagulation problems	4,577.25 €	3.94%
Arrhythmia	4,882.32 €	2.43%
Heart failure	2,291.03 €	2.35%
Anemia	5,195.95 €	1.93%
Psychiatric illness	2,616.89 €	1.62%
Cardiac arrest	4,806.45 €	1.45%
Pleural effusion	4,054.03 €	1.34%
Gastrointestinal bleeding	3,705.11 €	0.75%
Liver failure	3,239.83 €	0.73%
Pneumothorax	2,649.75 €	0.56%
Stroke	3,366.37 €	0.53%
Convulsions	2,796.47 €	0.34%
Cardiac ischemia	2,569.26 €	0.14%
Pancreatitis	10,116.47 €	0.03%
Endocarditis	5,561.40 €	0.03%
Meningitis	3,211.18 €	0.00%

Regarding demographic factors and previous comorbidities, except for asthma, all the variables were statistically significant, with a higher number of X-rays in men and patients over 60 years of age, and all the comorbidities analyzed in Table 4. The patients older than 60 years had a lower rate of CAT, as did patients with some previous comorbidity, such as hypertension or cardiovascular disease. Only patients with asthma had a higher rate of CAT.

**Table 4:** Relation between cost of diagnostic imaging and demographics and comorbidity variables.

		**RX**	**RX cost/per patient**	***P*-value**	**CAT**	**CAT cost/per patient**	***P*-value**
Gender							
	Male	1.36 ± 0.85	29.69 €	<0.001	0.08 ± 0.29	10.91 €	0.075
	Female	1.13 ± 0.78	24.70 €		0.10 ± 0.30	12.85 €	
Age							
	≤ 60 years	1.04 ± 0.80	22.71 €	<0.001	0.10 ± 0.31	13.05 €	0.030
	>60 years	1.40 ± 0.82	30.33 €		0.08 ± 0.28	10.52 €	
Actual smoker							
	No	1.29 ± 0.82	27.20 €	0.021	0.10 ± 0.29	11.78 €	0.131
	Yes	1.35 ± 0.81	29.47 €		0.12 ± 0.34	15.73 €	
Diabetes							
	No	1.21 ± 0.82	26.42 €	<0.001	0.10 ± 0.29	11.86 €	0.938
	Yes	1.46 ± 0.77	31.78 €		0.10 ± 0.31	12.41 €	
Hypertension							
	No	1.13 ± 0.84	24.77 €	<0.001	0.10 ± 0.31	13.02 €	0.034
	Yes	1.42 ± 0.75	31.05 €		0.08 ± 0.28	10.48 €	
Chronic cardiac diseases							
	No	1.21 ± 0.82	26.33 €	<0.001	0.09 ± 0.30	12.57 €	0.036
	Yes	1.45 ± 0.79	31.69 €		0.07 ± 0.28	9.65 €	
Asthma							
	No	1.26 ± 0.82	27.46 €	0.281	0.09 ± 0.29	11.60 €	0.027
	Yes	1.21 ± 0.87	26.37 €		0.14 ± 0.36	18.25 €	
Chronic bronchitis							
	No	1.26 ± 0.82	27.13 €	<0.001	0.09 ± 0.30	12.06 €	0.722
	Yes	1.41 ± 0.72	30.73 €		0.09 ± 0.31	11.49 €	

## Discussion

Our study aimed to estimate the costs to the health system derived from health care for COVID-19 disease in primary care and hospital care. For this purpose, we performed an estimation on a large sample of patients with COVID-19, 3,525 patients, during the inclusion period, which allowed us to have confidence in the results obtained. Our findings show an average of €4294.36 of expenditure on hospital stay and €14440 of expenditure on ICU stay. We only found similar data in studies carried out by Osakidetza (11) where the hospital cost was calculated at €8,500 and €33,400, respectively. In our particular case, the data we estimate are only for the length of stay; if the imaging diagnosed tests performed on patients, biochemical analyses, treatments, and management of complications that appear during admission were added, the expenditure would be similar. On the other hand, our study provides an estimate of the cost of the COVID-19 process in primary health care, of which we are not aware of any previous cost study.

We can find some articles that try to relate the poor health management of the pandemic with “low” public spending in our country, as in other European countries, without finding a significant association between the poor coordination of primary and specialized care and poor governmental management (12). Some studies highlight other factors that may be associated with poor clinical outcomes such as social inequalities, inequalities in health care due to environmental or demographic factors, population aging, or vulnerability (13). Although there is evidence of the existence of an association between higher health expenditure and better health outcomes (14, 15), in our country, we have estimated the average expenditure per patient infected by the SARS-CoV-2 virus at around €2373.24, a figure that is increased by drugs or treatments, whether antivirals, antipyretics, analgesics, or management of complications of the infection. However, the data we provide reference the first wave of the pandemic and we believe that they could have been significantly lower due to the greater knowledge generated about the virus during the first months of the pandemic in our country. Nowadays, to advance the knowledge of the cost of COVID-19 in our country, we have shown the cost in health, but the direct cost of infection due to SARS-CoV-2 includes not only direct management of the disease but we also have to consider other expenditures, such as a temporary labor force adjustment plan, investment in preventive interventions and resources and treatment of COVID-19, and the long-term health consequences of the disease (respiratory complications, neurological or cardiovascular diseases, persistent mental health problems, etc.).

On the other hand, complications in patients with COVID-19 generate additional costs concerning the lack of development. The most frequent complications in our study have been kidney, respiratory, and cardiovascular-hematological issues, which is in line with other hospital studies (16). Kidney failure appears in 8.46% of patients, being the most frequent and assuming by itself an extra cost of around 100%, but respiratory complications as a whole (respiratory infection, acute respiratory distress syndrome, and pneumonia) account for more than 18%, and although they do not all appear continuously in all patients, they can lead to cost increases of between €2625.36 (in the most favorable case) and €9485.33 (in the most unfavorable situation). This represents a cost increase of more than 200%. The same can happen with cardiovascular and hematological alterations, which will greatly increase the extra costs. Hematological alterations up to more than 300% in the most unfavorable case, anemia and coagulopathies (€9773.2), and up to 600% in the case of cardiac and circulatory complications (between €2291.03 and €23476.83). We show how the complication that increases costs the least, heart failure, represents a 50% increase in the costs of a hospitalized patient without serious complications. It has not been possible to retrieve articles that account for the costs of complications, so these data are valuable when making a first approximation of certain additional costs for complications of COVID-19.

Knowing the costs derived from some chronic pathologies is of great interest since it allows us to compare the costs with general care. In this sense, all the variables studied have shown a statistically significant relationship to some of the outcome variables, such as the performance of X-rays and CAT scans, in addition to gender and age. It would be interesting to know in the field of primary care how having chronic diseases affects the cost of the process, since most of the management of chronic processes, including complex chronic ones, is carried out in community settings (17).

Concerning the variable smoking, there are strong data from studies in China that support the increased risk of COVID-19 pneumonia in smokers (18). This would be a key explanatory factor for the increase in costs in these patients. In our study, we observed a significant increase in the number of X-rays, which is related to the greater severity of the processes described in the antecedents, which agrees with the data of our study since the most frequent complications are respiratory and consequently, an increase in costs. Respiratory conditions, closely related to smoking, also represent an increase in the consumption of imaging tests. This is important because various studies have found that up to 18.5% of those hospitalized for COVID-19 were smokers (19), which allows us to deduce that this fact will increase the costs of hospitalizations.

In people with diabetes, it is known that the cost of average hospital admission in people with type II diabetes is €25,018 if they have good control, and €57,244 if there is poor control (20). This is a striking fact given that some studies estimate the cost of admission in non-diabetic people at €16,993 and €13,908, (20) approximate figures for the study of the Basque Country (11), and ours if we impute additional costs to the hospital stay. We have been able to observe how in our study, being diabetic significantly increases the number of X-rays, which partially justifies the fact that the costs are higher in these patients.

In people with hypertension, we observe the same phenomenon: significance has been found in the consumption of resources and costs, as it affects X-ray and CAT scan consumption. The same is observed in heart diseases.

## Conclusions

While the cost of a patient with COVID-19 without hospitalization is €729.79, the costs of a hospitalized patient are between €4294.36 and €14440.68, if there is ICU admission. Despite the difficulty in obtaining exact costs, complications greatly increase treatment costs, the most frequent complications being kidney failure, respiratory, cardiovascular, and hematological complications. The chronic pathologies studied have made it possible to conclude that in most cases, the consumption of radiological imaging services, X-ray and CAT scan, increases significantly the costs.

## Limitations

The present study supposes an approximation to the costs and extra costs of the processes in COVID-19 management both with hospitalization and without hospitalization. However, it is not possible to carry out an exact calculation with the available data, since it does not count costs that influence the processes, such as pharmacological treatments or the consumption of human resources. Another limitation is that of the study design, observational and descriptive, which does not allow absolute certainty in the relationships between the variables studied.

## Data Availability Statement

The raw data supporting the conclusions of this article will be made available by the authors, contacting with the corresponding author.

## Ethics Statement

The studies involving human participants were reviewed and approved by Clinical Research Ethics Committee of LPUH, Madrid, with LPUH code: PI-4155. Written informed consent for participation was not required for this study in accordance with the national legislation and the institutional requirements.

## Author Contributions

EA-E conceived and designed the experiments, analyzed the data, prepared figures and/or tables, and approved the final draft. MQ-D performed the experiments, prepared figures and/or tables, and approved the final draft. JC-M conceived and designed the experiments, analyzed the data authored or reviewed drafts of the article, and approved the final draft. MD-R performed the experiments, analyzed the data, authored or reviewed drafts of the article, and approved the final draft. BO-B conceived and designed the experiments, authored or reviewed drafts of the article, and approved the final draft. RM-B performed the experiments, authored or reviewed drafts of the article, and approved the final draft. IS-A analyzed the data, prepared figures and/or tables, and approved the final draft. RJ-V and CR-T conceived and designed the experiments, performed the experiments, prepared figures and/or tables, and approved the final draft. VG-C and AC-R performed the experiments, analyzed the data, prepared figures and/or tables, and approved the final draft. All authors contributed to the article and approved the submitted version.

## Conflict of Interest

The authors declare that the research was conducted in the absence of any commercial or financial relationships that could be construed as a potential conflict of interest.

## Publisher's Note

All claims expressed in this article are solely those of the authors and do not necessarily represent those of their affiliated organizations, or those of the publisher, the editors and the reviewers. Any product that may be evaluated in this article, or claim that may be made by its manufacturer, is not guaranteed or endorsed by the publisher.

## Footnotes

^1^ https://www.worldometers.info/coronavirus/country/spain/
